# Identification and targeted disruption of the mouse gene encoding ESG1 (PH34/ECAT2/DPPA5)

**DOI:** 10.1186/1471-213X-6-11

**Published:** 2006-02-28

**Authors:** Hisayuki Amano, Ken Itakura, Masayoshi Maruyama, Tomoko Ichisaka, Masato Nakagawa, Shinya Yamanaka

**Affiliations:** 1 Department of Stem Cell Biology, Institute for Frontier Medical Sciences, Kyoto University, Kyoto, Japan; 2 CREST, Japan Science and Technology Cooperation, Kawaguchi, Japan; 3 Department of Molecular Biology and Biochemistry, Osaka University Graduate School of Medicine/Faculty of Medicine, Suita, Japan

## Abstract

**Background:**

Embryonic stem cell-specific gene (*ESG*) 1, which encodes a KH-domain containing protein, is specifically expressed in early embryos, germ cells, and embryonic stem (ES) cells. Previous studies identified genomic clones containing the mouse *ESG1 *gene and five pseudogenes. However, their chromosomal localizations or physiological functions have not been determined.

**Results:**

A Blast search of mouse genomic databases failed to locate the *ESG1 *gene. We identified several bacterial artificial clones containing the mouse *ESG1 *gene and an additional *ESG1*-like sequence with a similar gene structure from chromosome 9. The ESG1-like sequence contained a multiple critical mutations, indicating that it was a duplicated pseudogene. The 5' flanking region of the *ESG1 *gene, but not that of the pseudogene, exhibited strong enhancer and promoter activity in undifferentiated ES cells by luciferase reporter assay. To study the physiological functions of the *ESG1 *gene, we replaced this sequence in ES cells with a β-geo cassette by homologous recombination. Despite specific expression in early embryos and germ cells, *ESG1*^-/- ^mice developed normally and were fertile. We also generated *ESG1*^-/- ^ES cells both by a second independent homologous recombination and directly from blastocysts derived from heterozygous intercrosses. Northern blot and western blot analyses confirmed the absence of ESG1 in these cells. These ES cells demonstrated normal morphology, proliferation, and differentiation.

**Conclusion:**

The mouse *ESG1 *gene, together with a duplicated pseudogene, is located on chromosome 9. Despite its specific expression in pluripotent cells and germ cells, ESG1 is dispensable for self-renewal of ES cells and establishment of germcells.

## Background

Embryonic stem (ES) cells were first derived from the blastocysts of mice in 1981 [1,2] and humans in 1998 [3]. ES cells have two important properties: theability to maintain pluripotency, which is the ability to differentiate into a wide variety of cells, and rapid proliferation. These characteristics make mouse ES cells an essential component of gene targeting technology. These qualitiesalso make human ES cells attractive sources for cell transplantation therapy to treat various diseases, including spinal cord injuries and juvenile diabetes. The molecular mechanisms underlying the pluripotency and rapid proliferation of ES cells are currently a major focus of the field of stem cell biology [4-6].

To identify molecules essential in ES cells for these properties, several groups have utilized transcriptome analyses to identify genes specifically expressed in ES cells and early embryos. These analyses, including DNA microarray analyses [7] and expressed sequence tag analyses [8-12], identified several common transcripts, including *ESG1 *that was also designated *dppa5 *or *ECAT2*.

ESG1 was originally identified as a transcript Ph34 that was down-regulated by retinoic acid in embryonic carcinoma cells [13]. The expression of this gene was confined in mice to early embryos and germ cells [14]. It is also expressed in pluripotent cells, including ES cells, embryonic germ cells, and multipotent germline stem cells [15]. *ESG1 *encodes a polypeptide of 118 amino acids that contains a single KH domain, which is an RNA-binding domain [16]. It remains unclear, however, if ESG1 functions as an RNA-binding protein or the roles it plays in ES cells and mice.

Previous genomic library screening by identified genomic clones containing the mouse *ESG1 *gene and seven pseudogenes [17]. Two of these pseudogenes exhibit a similar exon-intron structure as the *ESG1 *gene, indicating their generation by gene duplication. The five remaining pseudogenes did not contain any introns, indicating that these were generated by retrotransposition of *ESG1 *transcripts. The chromosomal localizations of the mouse *ESG1 *gene and pseudogenes, however, have not been reported.

In this study, we determined the structure of the mouse gene encoding this protein and related pseudogenes. We also performed gene targeting to determine the physiological function of ESG1.

## Results and discussion

### Chromosomal localization and structures of mouse *ESG1 *gene and psedogenes

**Figure 1:**
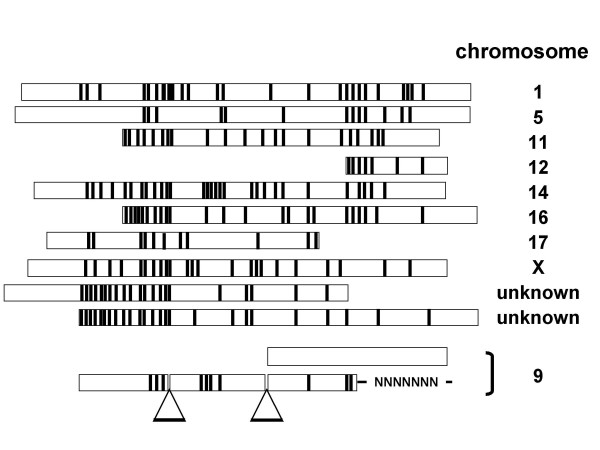
***ESG1*pseudogenes identified by a Blast search of mouse genomic databases**. Substitution mutations are indicated by black lines. Intron-like gap sequences are indicated with triangles. Chromosomal localizations are shown on the right.

To determine the chromosomal localizations of the mouse *ESG1 *gene and pseudogenes, we performed a Blast analysis of the mouse genomic database with the *ESG1 *cDNA sequence as a query. We identified several putative pseudogenes without introns on chromosomes 1, 5, 11, 12, 14, 16, 17, and X (Figure 1). In addition, two intron-less pseudogenes were identified in DNA fragments for which the chromosomal localization remained unmapped. While these pseudodgenes have significant homology to *ESG1 *cDNA, they could not produce functional proteins, because of critical mutations. This result suggests that there are a larger number of intron-less pseudogenes than previously anticipated. Existence of multiple retropseudogenes is a hallmark of pluripotent cell-specific genes [18].

On chromosome 9, we identified a DNA fragment similar to the *ESG1 *gene that included two putative introns. These putative first and second exons, however, contained (4) multiple mutations of the *ESG1 *cDNA sequence. The putative third exon was identical to that of the previously reported *ESG1 *gene. Also on chromosome 9, we identified another DNA fragment that was similar, but not identical, to the third exon of the ESG1 gene. These findings suggest that these *ESG1*-like sequences on chromosome 9 have not been correctly assembled.

**Figure 2:**
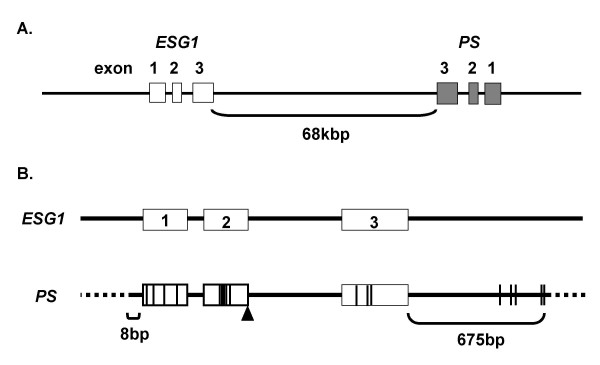
**BAC clones containing the *ESG1 *gene and a duplication pseudogene (PS)**. A) Localization of the gene and PS on chromosome 9. B) Sequence comparison of the gene and PS revealed that these are homologous from eight base pairs upstream of the first exon to 675 bp downstream of the third exon. Substitution mutations are indicated by black lines. The insertion is indicated by an open triangle.

**Figure 3:**
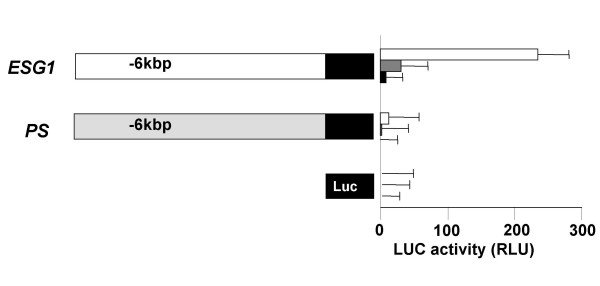
**Promoter/enhancer activity of the *ESG1 *gene and pseudogene**. DNA fragments of ~6 kbp isolated from the 5' flanking regions of the gene and PS were transferred into luciferase reporter plasmids. We introduced the reporter genes into undifferentiated ES cells (open columns), retinoic acid-treated ES cells (grey columns), and NIH3T3 cells (closed columns). Data represent the averages and standard deviations of three independent experiments.

To obtain DNA fragments containing the *ESG1 *gene, we screened the bacterial artificial chromosome (BAC) DNA pool by PCR using primer pairs that would only amplify the real gene, not the pseudogenes. We obtained two independent, but overlapping BAC clones. Southern blot analyses and sequencing demonstrated that these clones contained a sequence exhibiting complete identity with *ESG1 *cDNA that was interrupted by two putative introns (Figure 2A). The two intron sequences begin with GT and terminate with AG, fulfilling the GT-AG rule of exon-intron junctions [19]. The 5' flanking region of this DNA fragment exhibited strong promoter/enhancer activity by luciferase reporter assays in undifferentiated ES cells, but not in somatic cells (Figure 3). The same fragment showed much weaker activity after induction of differentiation by retinoic acid. We concluded that this sequence is the *ESG1 *gene.

We also found that the two BAC clones contained another *ESG1*-like sequence (Figure 2A). The two sequences, separated by a 68 kbp intergenic sequence, were oriented in opposite directions. The *ESG1*-like sequence exhibited greater than 95% identity to the exons and introns of the *ESG1 *gene. This sequence, however, contained critical nucleotide substitutions in all of the exons and one nucleotide insertion in exon 2 (Figure 2B). Although 675 base pairs of the 3' flanking regions were conserved between the ESG1 gene and the pseudogene, only five base pairs of the 5' flanking region were identical. This 5' flanking region (~6 kbp) did not possess any promoter/enhancer activity in luciferase reporter assays (Figure 3). It is thus unlikely that this sequence is transcribed or translated into a functional protein. This sequence likely represents a duplication pseudogene. Bierbaum previously reported the existence of two pseudogenes with similar exon-intron organization as the *ESG1 *gene [17]. We could not determine which of these two pseudogenes corresponds to the one we identified or the location of the remaining pseudogene.

### Targeted disruption of the mouse *ESG1 *gene

**Figure 4:**
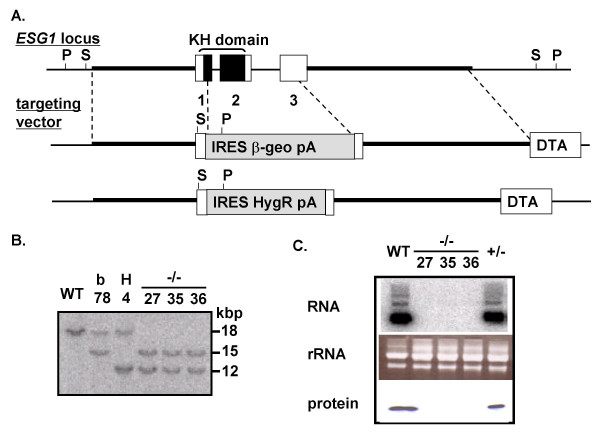
**Targeted disruption of the mouse ESG1 gene**. A) Targeting strategy. Homologous regions are indicated by thick lines. Recognition sites of *Pst*I (P) and *Spe*I (S), which were used for Southern blot analyses, are shown. The gene encoding diphtheria toxin A (DTA) was inserted at the 3' end of the targeting vectors to facilitate negative selection. B) Southern blot analyses confirming homologous recombination. WT, wild-type ES cells; β, β-geo +/- ES cells; H, HygR +/- ES cells; -/-, ESG1-null ES cells. Numbers indicate clone numbers. C) Northern blot (upper) and western blot (lower) analyses of wild-type ES cells (WT), ESG1-null ES cells (-/-, three clones) and heterozygous ES cells (+/-). Northern blot was performed as previously described [20]. To confirm the loading of equal amounts of RNA, ethidium bromide staining of ribosomal RNA is also shown (middle).

To study the function of ESG1, we deleted the gene by homologous recombination in mouse ES cells. We replaced the three exons with either a fusion of the neomycin-resistance and β-galactosidase genes (β-geo) or the hygromycin resistant gene (HygR) using two targeting vectors (Figure 4A) introduced into RF8 ES cells by electroporation. We obtained eight ES cell clones with correct homologous recombination of the β-geo targeting vector, which was confirmed by Southern blot analysis (Figure 4B). We obtained only one clone with correct homologous recombination of the HygR targeting vector.

To obtain homozygous mutant ES cells, we introduced the β-geo vector into HygR heterozygous ES cells. Of 105 G418-resistant colonies tested, 49 were homozygous for *ESG1 *deletion. Northern blot and western blot analyses confirmed the absence of ESG1 in these cells (Figure 4C). In 29 clones, the β-geo vector had replaced the HygR vector, such that the cells remained heterozygous. In the remaining 27 clones, the β-geo vector was integrated at non-homologous sites.

**Figure 5:**
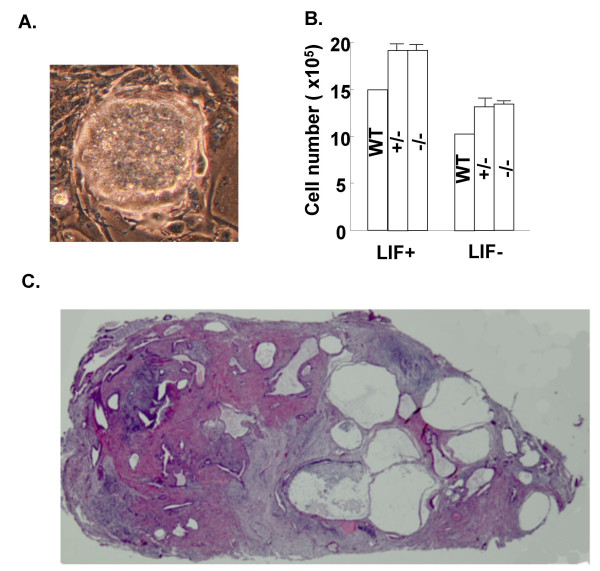
**Analyses of ESG1-null ES cells**. A) The morphology of ESG1-null ES cell colonies grown on STO feeder cells. B) Growth curve of wild-type (WT), ESG1-null (-/-) and heterozygous (+/-) ES cells. Each clone (1 × 10^4 ^cells/well) was plated in 24-well plates. Cell numbers were determined with a Coulter counter at 2, 4, and 6 days. Data of +/- and -/- cells are shown as averages and standard deviations of three independent clones. C) A section of teratoma derived from ESG1-null ES cells (hematoxylin & eosin staining).

*ESG1*^-/- ^ES cells exhibited normal morphology (Figure 5A). These cells also proliferated at a speed comparable to that of the control (heterozygous and wild-type) cells (Figure 5B). *ESG1*^-/- ^cells differentiated normally after the removal of leukemia inhibitory factor (Figure 5B) or retinoic acid treatment (not shown). When transplanted into hind flanks of nude mice, these cells produced teratomas, tumors containing components of all three germ layers (Figure 5C). These results indicate that ESG1 is dispensable for the self-renewal properties and pluripotency of ES cells.

**Figure 6:**
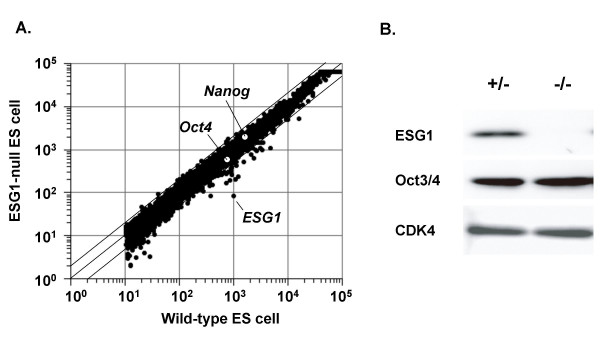
**Gene expression analyses of ESG1-null ES cells**. A) DNA microarray analyses. Total RNA from wild-type ES cells and ESG1-null ES cells were labeled with Cy3 and Cy5, respectively. Samples were hybridized to Agilent Mouse Developmental Microarrays. The averages of two independent clones are shown. B) Western blot analyses. Cell lysates from ESG1^+/- ^and ESG1^-/- ^cells were examined for expression of ESG1, Oct3/4 and CDK4 with immunoblotting.

We examined the gene expression profiles of *ESG1*^-/- ^ES cells using oligonucleotide-based DNA microarrays representing ~20,000 genes. In comparison to control ES cells, *ESG1 *was identified as the gene reduced to the greatest extent in *ESG1*^-/- ^ES cells (Figure 6A). The expression of ES cell marker genes, such as *Nanog *and *Oct3/4*, was normal in *ESG1*^-/- ^ES cells. We confirmed normal Oct3/4 expression at protein levels by Western blot (Figure 6B). The overall gene expression profiles were similar between control ES cells and *ESG1*^-/- ^ES cells. Several genes exhibited a greater than two-fold reduction in *ESG1*^-/- ^cells, including *Krt1-8, Pem, Ctgf, Ptgs2, Igf2 and Inhba*. These genes might be regulated directly or indirectly by ESG1. Since ESG1 contains a KH-type RNA-binding domain, it may stabilize mRNA of these genes. Further studied are required to clarify this possibility.

To generate ESG1-knockout mice, we injected β-geo-*ESG1*^+/- ^ES cell clones into the blastocysts of C57BL6 mice. We obtained germline transmission from three clones. We obtained *ESG1*^-/- ^mice at the Mendelian ratios (36 wild-type, 69 *ESG1*^+/-^, and 45 *ESG1*^-/-^) from intercrosses of *ESG1*^+/- ^mice. These animals exhibited normal development, gross appearance, and fertility (not shown). Histological examination of testis and ovary could not identify any abnormalities (not shown). These data demonstrated that ESG1 is dispensable for both mouse development and germ cell formation.

We also generated ES cells from blastocysts obtained by intercrosses of *ESG1*^+/- ^males and *ESG1*^-/- ^females. Of the eight ES cell lines established, two clones were *ESG1*^-/-^. These ESG1-null ES cells demonstrated normal morphology, proliferation, and differentiation (not shown), confirming that ESG1 is dispensable in ES cells.

## Conclusion

To analyze the physiological roles of ESG1, we identified the mouse gene on chromosome 9 and deleted it by homologous recombination in ES cells. Despite specific expression in early embryos, germ cells, and pluripotent cells, our data demonstrated that ESG1 is dispensable for mouse development, germ cell formation, and ES cell self-renewal.

## Methods

### Identification and analyses of BAC clones containing the mouse *ESG1 *gene

To identify bacterial artificial chromosome (BAC) clones containing mouse *ESG1 *gene, we performed PCR-based screening of mouse BAC library DNA pools (Research Genetics) using the pH34-u38 (5'-GAAGTCTGGTTCCTTGGCAGG-3') and pH34-L394 (5'-ACTCGATACACTGGCCTAGC-3') primers. Following restriction enzyme digestion, we performed Southern blot analyses of BAC clones as described [20] using the pH34-U258 (5'-CTCGAGTGTACAGTCAAGTGGTTGCTGGGA-3'), pH34-U65 (5'-GTGACCCTCGTGACCCGTAA-3'), pH34-intron1L (5'-CTGCGTGAGAGAAACACCAAACAGGC-3'), pH34-L545 (5'-TGTGAATGGGAAGGTTACCACTCT-3') and pH34-SCL1 (5'-GCCCTCTTCTGGTTTGTCTCGAAAT-3') probes. Hybridization with these probes revealed bands containing either the *ESG1 *gene or pseudogenes.

To sequence the region containing the mouse *ESG1 *gene and the 3' flanking region, we subcloned a ~15 kbp *Xho*I-*Sal*I fragment into the pZERO-2 vector (Invitrogen). *Hind*III- or *EcoR*I-digested fragments of this vector were then cloned into pBluescript KS(-) for sequencing. To sequence the *ESG1 *pseudogene and the 3' flanking region, an 8 kbp *Not*I/*Xho*I fragment was cloned into pBluescript KS(-). *BamH*I- or *Pst*I- fragments of this vector were also cloned into pBluescript KS(-). To identify the sequence containing the 5' flanking regions of the *ESG1 *gene and the related pseudogenes, we used a TOPO walker kit (Invitrogen) with the pH34-T2L (5'-ACTAGTCGCAGCAGGGATCCAGGAATATCT-3') and pH34-L394 primers. The resulting sequence was cloned into pCR2.1 (Invitrogen). We obtained a ~6 kbp band from the *Nsi*I-digensted library; *Xba*I-, *Spe*I-, *EcoR*I-, and *Pst*I-digested fragments of this band were cloned into pBluescript KS(-) for sequencing. This fragment contained the 5' flanking region of the *ESG1 *gene. A ~3 kbp fragment, obtained from the *Sac*I-digested library, was cloned into pCR2.1 for sequencing. This fragment was contained the 5' region flanking the pseudogene.

### Construction of *ESG1 *targeting vectors

We replaced all of the *ESG1 *exons with two targeting vectors containing either an IRES-β-geo cassette [21] or an IRES-HygR cassette by promoter trap selection. We amplified the 5' arm (1.8 kbp) using KOD plus (TOYOBO) with the pH34-targetpair5-U (5'-CCGCGGAAAGTCAAGAGATTGGGTGG-3') and pH34-targetpair5-L (5'-GCGGCCGCCTTTACGGGTCACGAGGGTCAC-3') primers. The 3' arm (5.8 kbp) was amplified using the pH34-targetpair3-U (5'-TGTGGCCAGTGTTTGGTTCTGGCGGG-3') and pH34-targetpair3-L (5'-CTCGAGGACTCGCCATTCTAGCCAAG-3') primers. The IRES β-geo or IRES HygR cassettes were ligated in between the two PCR fragments. The diphtheria toxin A cassette was placed downstream of the 3' arm. After linearization with *Sac*II, these targeting vectors were electroporated into 2.0 × 10^7 ^RF8 ES cells [22] using a Gene pulser (BIORAD). Transfected cells were selected with 250 μg/mL G418 or 100 μg/mL hygromycin B, respectively. Genomic DNA from G418- or hygromycin B-resistant colonies was screened for homologous recombination by Southern blotting.

### Southern blot screening for homologous recombination

ES cells genomic DNA was extracted with PUREGENE™ Cell Lysis Solution (Gentra systems). For 5' Southern blot analysis, genomic DNA was first digested with *Pst*I, then separated on an 0.8% agarose gel and transferred to a nylon membrane as described [20]. A 560 bp 5' probe was amplified using the ESG1S5 (5'- GATGGTGGTGGTGACTCAGAG -3') and ESG1AS5 as (5'- CCTCCATTGCCTCTATATCAG -3') primers. The probe specifically labeled an 18 kbp band from the wild-type locus, a 15 kbp band from the β-geo locus, and a 12 kbp band from the HygR locus. Genomic DNA was also digested with SpeI for 3' Southern blot analysis. A 1,010 bp 3' probe was amplified with the pH34U-8000 (5'- CCAACCAGCCAGAGTTTCAGTTAT -3') and pH34L-9000 (5'-GATAAGCTGCTGCCAAAAGACAAG -3') primers. The probe hybridized to an 11.5 kbp band from the wild-type locus, a 12.5 kbp band from the β-geo locus, and a 9.5 kbp band from the HygR locus.

### Generation of anti-ESG1 polyclonal antibodies

The coding sequence of *Esg1 *was amplified by PCR with the pH34-gw-s (5'- AAAAAGCAGGCTGGATGATGGTGACCCTCGTGA-3') and pH34-gw-as (5'- AGAAAGCTGGGTCTGCATCCAGGTCGGAGACA-3') primers. To construct pDONR-pH34, the resulting PCR product was subcloned into pDONR201 (Invitrogen). pDONR-pH34 was interacted with pDEST17 (Invitrogen) by LR recombination. After introduction of the resulting expression vector pDEST17-pH34 into BL21-AI *E. coli *(Invitrogen), recombinant protein production was induced according to the manufacture's protocol. Histidine-tagged ESG1 was purified using Ni-nitrilotriacetic acid agarose (Qiagen) under denaturing conditions in the presence of 8 M urea. After dialysis against 6 M urea, the recombinant proteins were injected into New Zealand White rabbits to generate anti-ESG1 polyclonal antibodies.

### Western blot

After preparation of ES cell extracts with M-Per (Pierce), cellular proteins were separated on sodium dodecyl sulfate (SDS)-14% polyacrylamide gels and transferred to nitrocellulose membranes (Millipore). Membranes were incubated with anti-ESG1 (1/500 dilution), anti-Oct3/4 (1/500; Santa Cruz Biotechnology), anti-CDK4 (1/200; Santa Cruz Biotechnology), and anti-GFP (1/1000; MBL) primary antibodies. Horseradish peroxidase-conjugated anti-rabbit and anti-mouse immunoglobulins (1/5000; Cell Signaling) were used to detect antibody binding. We visualized bound antibody with an ECL Western Blotting Detection System (Amersham).

### Derivation of ESG1-deficient ES cells from blastocysts

*Esg1*^+/-^*or ESG1*^-/- ^mutant female mice were injected with Tamoxifen (10 μg) and Depo-provera (1 mg) subcutaneously on the third day of pregnancy. Four days later, embryos in diapause were flushed out of the uterus and cultured on STO feeder cells in four-well plates in Dulbecco's Modified Eagle Medium (DMEM) supplemented with 20% Fetal Bovine Serum (Hyclone), 0.1 mM Non-Essential Amino Acids (Invitrogen), 2 mM L-glutamine (Invitrogen), 50 U/ml Penicillin-Streptomysin (Invitrogen), and 0.11 mM 2-mercaptoethanol (Invitrogen). After six days, the central mass of each explant was harvested, rinsed in PBS, and placed in a drop of trypsin for a few minutes. The cell mass was collected with a finely drawn-out Pasteur pipette preloaded with medium, ensuring minimal carryover of the trypsin. The cells were gently transfered into a fresh well with 20% FBS-containing medium. The resulting primary ES cell colonies were individually passaged into wells of four-well plates containing STO feeder cell layers. Thereafter, cells were expanded by trypsinization of the entire culture.

### Microarrays

Total RNA from wild-type ES cells and ESG1^-/- ^ES cells was labeled with Cy3 and Cy5, respectively. The samples were hybridized to a Mouse Development Microarray (Algilent) according to the manufacturer's protocol. Arrays were scanned with a G2565BA Microarray Scanner System (Agilent). Hybridization was repeated with two independent clones. Data were analyzed with GeneSprings software (Silico Genetics).

## Authors' contributions

HA carried out the phenotypic studies of ESG1 knockout mice. KI determined the chromosomal localizations of the ESG1 gene and pseudogenes and constructed the targeting vector. TI carried out mouse embryo manipulation. MM and MN carried out ES cell culture. SY conceived of the study, and participated in its design and coordination and helped to draft the manuscript. All authors read and approved the final manuscript.

## References

[B1] Evans MJ, Kaufman MH (1981). Establishment in culture of pluripotential cells from mouse embryos. Nature.

[B2] Martin GR (1981). Isolation of a pluripotent cell line from early mouse embryos cultured in medium conditioned by teratocarcinoma stem cells. Proc Natl Acad Sci U S A.

[B3] Thomson JA, Itskovitz-Eldor J, Shapiro SS, Waknitz MA, Swiergiel JJ, Marshall VS, Jones JM (1998). Embryonic stem cell lines derived from human blastocysts. Science.

[B4] Chambers I, Smith A (2004). Self-renewal of teratocarcinoma and embryonic stem cells. Oncogene.

[B5] Wobus AM, Boheler KR (2005). Embryonic stem cells: prospects for developmental biology and cell therapy. Physiol Rev.

[B6] Boiani M, Scholer HR (2005). Regulatory networks in embryo-derived pluripotent stem cells. Nat Rev Mol Cell Biol.

[B7] Tanaka TS, Kunath T, Kimber WL, Jaradat SA, Stagg CA, Usuda M, Yokota T, Niwa H, Rossant J, Ko MS (2002). Gene expression profiling of embryo-derived stem cells reveals candidate genes associated with pluripotency and lineage specificity. Genome Res.

[B8] Bortvin A, Eggan K, Skaletsky H, Akutsu H, Berry DL, Yanagimachi R, Page DC, Jaenisch R (2003). Incomplete reactivation of Oct4-related genes in mouse embryos cloned from somatic nuclei. Development.

[B9] Tokuzawa Y, Kaiho E, Maruyama M, Takahashi K, Mitsui K, Maeda M, Niwa H, Yamanaka S (2003). Fbx15 is a novel target of Oct3/4 but is dispensable for embryonic stem cell self-renewal and mouse development. Mol Cell Biol.

[B10] Takahashi K, Mitsui K, Yamanaka S (2003). Role of ERas in promoting tumour-like properties in mouse embryonic stem cells. Nature.

[B11] Mitsui K, Tokuzawa Y, Itoh H, Segawa K, Murakami M, Takahashi K, Maruyama M, Maeda M, Yamanaka S (2003). The Homeoprotein Nanog Is Required for Maintenance of Pluripotency in Mouse Epiblast and ES Cells. Cell.

[B12] Maruyama M, Ichisaka T, Nakagawa M, Yamanaka S (2005). Differential roles for sox15 and sox2 in transcriptional control in mouse embryonic stem cells. J Biol Chem.

[B13] Astigiano S, Barkai U, Abarzua P, Tan SC, Harper MI, Sherman MI (1991). Changes in gene expression following exposure of nulli-SCCl murine embryonal carcinoma cells to inducers of differentiation: characterization of a down-regulated mRNA. Differentiation.

[B14] Western P, Maldonado-Saldivia J, van den Bergen J, Hajkova P, Saitou M, Barton S, Surani MA (2005). Analysis of Esg1 Expression in Pluripotent Cells and the Germline Reveals Similarities with Oct4 and Sox2 and Differences Between Human Pluripotent Cell Lines. Stem Cells.

[B15] Kanatsu-Shinohara M, Inoue K, Lee J, Yoshimoto M, Ogonuki N, Miki H, Baba S, Kato T, Kazuki Y, Toyokuni S, Toyoshima M, Niwa O, Oshimura M, Heike T, Nakahata T, Ishino F, Ogura A, Shinohara T (2004). Generation of pluripotent stem cells from neonatal mouse testis. Cell.

[B16] Gibson TJ, Thompson JD, Heringa J (1993). The KH domain occurs in a diverse set of RNA-binding proteins that include the antiterminator NusA and is probably involved in binding to nucleic acid. FEBS Lett.

[B17] Bierbaum P, MacLean-Hunter S, Ehlert F, Moroy T, Muller R (1994). Cloning of embryonal stem cell-specific genes: characterization of the transcriptionally controlled gene esg-1. Cell Growth Differ.

[B18] Pain D, Chirn GW, Strassel C, Kemp DM (2005). Multiple retropseudogenes from pluripotent cell-specific gene expression indicates a potential signature for novel gene identification. J Biol Chem.

[B19] Breathnach R, Chambon P (1981). Organization and expression of eucaryotic split genes coding for proteins. Annu Rev Biochem.

[B20] Yamanaka S, Zhang XY, Miura K, Kim S, Iwao H (1998). The human gene encoding the lectin-type oxidized LDL receptor (OLR1) is a novel member of the natural killer gene complex with a unique expression profile. Genomics.

[B21] Mountford P, Zevnik B, Duwel A, Nichols J, Li M, Dani C, Robertson M, Chambers I, Smith A (1994). Dicistronic targeting constructs: reporters and modifiers of mammalian gene expression. Proc Natl Acad Sci U S A.

[B22] Meiner VL, Cases S, Myers HM, Sande ER, Bellosta S, Schambelan M, Pitas RE, McGuire J, Herz J, Farese RVJ (1996). Disruption of the acyl-CoA:cholesterol acyltransferase gene in mice: evidence suggesting multiple cholesterol esterification enzymes in mammals. Proc Natl Acad Sci U S A.

